# Combating inconsistent evaluation of intra-tumor immune status by a novel transcriptomic signature in hepatocellular carcinoma

**DOI:** 10.1038/s41392-022-01262-x

**Published:** 2023-02-10

**Authors:** Linmeng Zhang, Ning Tang, Chen Yang, Haigang Geng, Hualian Hang, Wenxin Qin, Cun Wang

**Affiliations:** 1 State Key Laboratory of Oncogenes and Related Genes, Shanghai Cancer Institute, Renji Hospital, Shanghai Jiao Tong University School of Medicine, Shanghai, China; 2 Department of Gastrointestinal Surgery, Renji Hospital, School of Medicine, Shanghai Jiao Tong University, Shanghai, China; 3 Department of Liver Surgery, Renji Hospital, Shanghai Jiao Tong University School of Medicine, Shanghai, China

**Keywords:** Tumour heterogeneity, Tumour immunology

**Dear Editor**,

Harnessing the power of the immune system via immune checkpoint inhibition has revolutionized the treatment paradigm of many malignancies, including hepatocellular carcinoma (HCC).^1^ Despite encouraging efficacy seen with the immune checkpoint blockade (ICB) agents in a subset of patients with HCC, however, there remain a large number of HCC patients experience ICB resistance and failed to derive durable benefit from these agents, which underscores the need of patient selection for ICB treatment.^1^ Previous study showed that HCC tumors could be stratified into two classes: inflamed and non-inflamed classes.^2^ In contrast to non-inflamed class, inflamed class exhibits higher immune infiltration and cytolytic activity, and is associated with enhanced responsiveness to ICB therapy.^2,3^ Accordingly, determining the immune status of HCC tumors has important clinical significance, which can be leveraged to identify patients who are most likely to benefit from ICB therapy. Recently, a study by Montironi et al. reports a 20-gene signature that can be used to divide HCC tumors into these two classes.^4^ This signature was developed based on transcriptomic data from single-region samples, and has been demonstrated to have a favorable performance for immune status prediction.^4^

Extensive evidence has indicated the existence of intra-tumor heterogeneity (ITH) in HCC. Briefly, different intra-tumor regions can exhibit significant differences at multiple levels, including transcriptome, genome, epigenome, and proteome.^5–8^ Thus, it is reasonable to speculate that the evaluation of the overall immune status of HCC patient based on single region-derived transcriptomic data would not be reliable, unless the influence of ITH is appropriately addressed. Unfortunately, this factor was not considered during the development of 20-gene signature by Montironi et al., and thus this signature would likely yield inconsistent immune classification across different intra-tumor regions, which might limit its potential for clinical application. If this account is correct, there will be a need to develop novel signatures that can provide more robust prediction of the overall immune status when only single-region transcriptomic data is available. To verify above conjecture, here we have performed multi-region sampling on 14 patients enrolled in Renji hospital and collected a total of 75 multi-region samples. Besides, a novel algorithm has also been proposed to quantify gene-wise ITH, which has the potential to address the issue mentioned above (Fig. 1a).

**Fig. 1:**
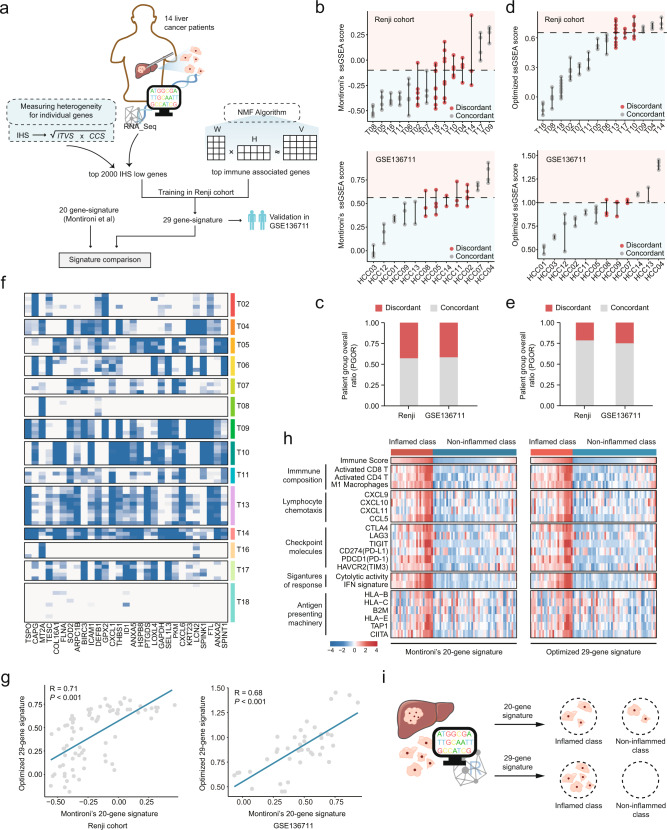
An optimized signature for immune evaluation through introducing multi-regional sequencing data into signature construction. **a** Schematic illustration of the study design. **b** The distribution of the signature scores of multi-regional tumor samples from Renji (upper) and GSE136711 (lower) cohort classified by 20-gene signature. Tumors were divided into inflamed and non-inflamed classes based on tertiles. Gray nodes represent the tumor samples from the same patient classified into a concordant classification. Red nodes represent the tumor samples from the same patient classified into discordant classifications. **c** The proportion of concordant and discordant classification in Renji and GSE136711 cohort classified by 20-gene signature. A concordant tumor means that the multi-region samples in this tumor all belong to a single class (inflamed or non-inflamed class), while a discordant tumor means that multi-region samples in this tumor belong to both inflamed and non-inflamed class. **d** The distribution of the signature scores of multi-regional tumor samples from Renji and GSE136711 cohort classified by optimized 29-gene signature. **e** The proportion of concordant and discordant classification in Renji and GSE136711 cohort classified by 29-gene signature. **f** Expression pattern of genes in the 29-gene signature in Renji cohort. **g** Correlation analysis of signature scores between 20-gene signature and optimized 29-gene signature in Renji and GSE136711 cohort. **h** Heatmap describing the molecular and immune characteristics of inflamed and non-inflamed HCC classes determined by 29-gene signature or 20-gene signature in Renji cohort. **i** Schematic diagram depicting the finding of the current study

We tested the robustness of Montironi’s 20-gene signature in our Renji cohort (Supplementary Table S1) and a public multi-region cohort (GSE136711 that includes 41 HCC tumors from 12 patients).^9^ The results showed that the Montironi’s signature might lead to inconsistent classifications in nearly 50% of patients with HCC in both multi-region cohorts (Fig. 1b, c). This result indicated that there was an urgent need to develop new predictive signatures that can be less influenced by ITH. To this end, we first devised a new metrics named integrated heterogeneity score (IHS) to quantify gene-wise heterogeneity (Supplementary Fig. 1a and Supplementary Table S2). Low-IHS genes show a clonal expression pattern within tumors and have a good representativeness of general expression (Supplementary Fig. 1b). A similar strategy to previous study was then adopted to generate inflamed signature, except that we only included low-IHS genes this time.^2^ Briefly, non-negative matrix factorization (NMF) analysis was performed to identify the immune-related expression module; the corresponding top-ranked genes (*n* = 500) based on their loadings were considered as immune-related genes. Then, these genes were intersected with top 2000 genes that had lowest IHS, leading to a 29-gene inflamed signature (Fig. 1a and Supplementary Table S3). Notably, this 29-gene signature has no genes common to previous 20-gene signature. However, functional similarity analysis suggested that most of genes in these two signatures had certain functional similarities, except for *GPX2* and *SPINT1* that might be less involved in the processes of immune response and thus had remarkably lower similarity scores (Supplementary Fig. 2).

The 29-gene signature exhibited stronger ability to concordantly classify HCC tumors than previous 20-gene signature, leading to discordances in less than 25% of cases in both cohorts (Fig. 1d, e). Also, it could be observed that genes in the new signature tend to have a clonal expression pattern (Fig. 1f and Supplementary Fig. 3A). Furthermore, preserving a strong correlation (Fig. 1g), the new signature can also efficiently classify HCC tumors into inflamed and non-inflamed classes, like the previous 20-gene signature (Fig. 1h and Supplementary Fig. 3B). The performance of the 29-gene signature in prognosis was also inspected. It could be observed that the 29-gene signature could hardly predict the overall survival outcome of patients with treatment-naïve HCC (Supplementary Fig. 4). This result was not surprising since this signature was developed for predicting the immune status rather than the prognosis of patients with HCC. Further, we tested whether this signature could inform the response to immunotherapy. Given that there are no public immunotherapy cohorts of HCC with transcriptomic data available at this moment, cohorts from other tumors are used as alternatives. It could be observed that our signature showed some predictive power for immunotherapeutic response in melanoma and urothelial cancer, which suggested that the potential of our 29-gene signature for clinical application in HCC merited further investigation (Supplementary Fig. 5).

The understanding of the roles of ITH in HCC has gradually advanced in recent years, and ITH has become a key factor that we must consider when studying the properties of tumors. To address the influence of ITH on immune status evaluation, we develop a new 29-gene signature that incorporates ITH information from multi-region sequencing data. In contrast to previously published signature, this new signature can offer a more reliable and robust evaluation of immune status of HCC patients who only have single-region transcriptomic profiles (Fig. 1i). Notably, although the new signature may present certain advantages over previous one, it still cannot overcome the inherent limitation in practical application. Specifically, liver biopsy is invasive and potentially risky, and is not routinely performed in clinical practice for HCC diagnosis. Thus, the tumor transcriptomic profiles that are necessary for determining the signature scores are unobtainable in a large proportion of HCC patients. Fortunately, the recently emerged approach based on liquid biopsy provides a feasible way to resolve this issue. In contrast to traditional tissue biopsies, liquid biopsy can be implemented more conveniently, less invasively, and without the limitation of sampling location and time. More importantly, this approach can be less influenced by the presence of ITH and has the potential to provide an accurate and unbiased assessment of tumor status.^10^

In this study, we demonstrated that the immune status evaluation using previous Montironi’s signature based on single-region samples might not be reliable due to the existence of ITH. A novel signature that can overcome ITH bias to some extent and provide a more concordant evaluation of inflamed status across different intra-tumor regions was thus proposed, offering new insight into the development of predictive biomarker for ICB therapy in HCC. However, it is necessary to emphasize that a comprehensive evaluation based on multi-region data would still be the most desirable approach to determine the overall immune status of patients with HCC.

## Supplementary information

Supplemental material

Supplementary Table S1

Supplementary Table S2

Supplementary Table S3

## Data Availability

All data included in this study are available upon request by contact with the corresponding author.
