# Supplementary material for: Combating inconsistent evaluation of intra-tumor immune status by a novel transcriptomic signature in hepatocellular carcinoma
Source: Signal Transduct Target Ther. 2023 Feb 10;8:61. doi: 10.1038/s41392-022-01262-x (PMC9911634; doi:10.1038/s41392-022-01262-x)
Supplement: Supplementary file 1 — Supplemental material [file 41392_2022_1262_MOESM1_ESM.docx]

Supplementary Materials for

**Combating inconsistent evaluation of intra-tumor immune status by a novel transcriptomic signature in hepatocellular carcinoma**

Linmeng Zhang^1, †^, Ning Tang^1, †^, Chen Yang^1, †^, Haigang Geng^2, †^, Hualian Hang^1, 3, *^, Wenxin Qin^1, *^, Cun Wang^1, 3, *^

1. State Key Laboratory of Oncogenes and Related Genes, Shanghai Cancer Institute, Renji Hospital, Shanghai Jiao Tong University School of Medicine, Shanghai, China.

2. Department of Gastrointestinal Surgery, Renji Hospital, School of Medicine, Shanghai Jiao Tong University, Shanghai, China.

3. Department of Liver Surgery, Renji Hospital, Shanghai Jiao Tong University School of Medicine, Shanghai, China.

† Linmeng Zhang, Ning Tang, Chen Yang and Haigang Geng contributed equally to this work.

Correspondence to Hualian Hang [hanghualian@shsmu.edu.cn](mailto:hanghualian@shsmu.edu.cn); Wenxin Qin: [wxqin@sjtu.edu.cn](mailto:wxqin@sjtu.edu.cn); Cun Wang [cwang@shsci.org](mailto:wxqin@sjtu.edu.cn)

**This PDF file includes:**

Materials and Methods

Figures. S1 to S5

Materials and Methods

**Patient samples**

Fourteen patients who underwent partial hepatectomy for primary HCC at Renji Hospital of Shanghai Jiao Tong University School of Medicine were included in this study. The inclusion criteria were as follows: age over 18 years at diagnosis, clinical and pathological diagnosis of HCC, no history of other malignancies, treatment-naïve prior to the surgery. This study was approved by the Ethics Committee of Renji Hospital (KY2021–114–B) and participants all have provided informed consents.

**RNA sequencing**

Total RNA was extracted from the frozen tissues by RNA mini kit (Qiagen, Hilden, Germany). Stranded mRNA-seq kit (NOVIZAN, China) was used to construct Poly-A enriched RNA-seq libraries. RNA sequencing was performed on Illumina NovaSeq 6000 platform with 40M pair-end 150bp reads per sample. The raw reads were mapped to hg38 reference genome using HISAT2 (version 2.2.1) with default parameters^1^. SubRead package was used to calculate gene-level read counts^2^. The resultant count data was normalized to transcript per million (TPM) for downstream analyses. Sequence data used in this study has been deposited at the National Omics Data Encyclopedia (NODE), which is hosted by the Shanghai Institutes for Biological Sciences, Chinese Academy of Sciences (Shanghai, China), under the accession code: OEP002956. GSE136711 is a public multi-region cohort, which included 41 HCC tumor samples from 12 patients^3^. Raw count data of GSE136711 were downloaded from GEO database and then normalized to TPM for downstream analyses.

**ITH quantification**

To quantitatively measure the gene-wise intra-tumor heterogeneity (ITH) level, we proposed a new computational strategy to calculate the integrated heterogeneity score (IHS) based on multi-regional samples from Renji cohort, which was integrated from variance-based and clustering-based approaches (**Supplementary Figure 1A**). For variance-based approach, linear mixed-effects analyses were used for the calculation of the intra-tumor variance (W = variance of differences within a tumor) and inter-tumor variance (B = variance of differences between patients) through the R package nlme^4, 5^. Higher intratumor variability score (ITVS) was associated with increased ITH level, with the formula as follows:

ITVS = $\frac{W}{W+B}$

Based on the concept that genes with low ITH could concordantly cluster tumor tissues extracted from the same patient, clustering-based approach was performed to cluster samples into sequentially increased groups using the hclust function in R^6, 7^. Patient group overall ratio (PGOR) was defined as follows:

PGOR = $\frac{N (patients grouped in the same clusters)}{N (total number of patients)}$

Using numerical integration, a curve based on PGOR could be obtained and the AUC of PGOR curve was calculated. Clustering concordance score (CCS) was then calculated as follows:

CCS = 1 $-\frac{AUC (PGOR)}{N \left( total number of patients \right)-1}$

The geometric mean of ITVS and CCS was defined as IHS, which ranged from 0 to 1. A low HIS indicated low gene-wise ITH level.

**Signature construction**

The non-negative matrix factorization (NMF) algorithm factorizes a data matrix A (n genes × m samples) into W and H matrixes^8^. W is an N (the number of genes) × k (the number of classes to be determined depends on the heterogeneity of the dataset) matrix, while H is a k × M (the number of experiments) matrix. This algorithm was employed to analyze the gene expression data of HCC samples in TCGA cohort. Briefly, immune enrichment score of each sample was first calculated using single sample gene set enrichment analysis (ssGSEA) based on the immune gene signature described by previous publication^9^. Then, NMF was performed to divide samples into different modules; we chose k = 5 as the number of factors, given that one of the modules could obtained the most significant immune enrichment score. This module was considered as ‘immune module’. Genes were ranked according to factor loading value of matrix W (immune factor weight) in immune module; the top-ranked genes (n = 500) were then taken as immune-related genes. The intersection of these immune-related genes and the top 2000 genes with lowest IHS yielded 29 genes. Subsequently, ssGSEA was applied to determine the signature scores of these genes^10^. The tumors with signature scores higher than the upper tertile was defined as inflamed class while others were defined as non-inflamed class^11, 12^.

**Public prognostic cohorts**

Six HCC cohorts with available survival information, including three sequencing-based cohorts (CHCC-HBV,^13^ LICA-FR^14^ and TCGA-LIHC^15^) and three microarray-based cohort (GSE14520,^16^ E-TABM-36^17^ and GSE54236^18^). Among the sequencing-based cohorts, LICA and LIHC provided raw counts, which were converted to transcript per million (TPM) for subsequent analyses,^19^ while CHCC only provided fragments per kilobase per million reads (FPKM) normalized data, which was also transformed into TPM. For microarray-based cohorts, normalized data was directly downloaded from the Gene Expression Omnibus (GEO) or the ArrayExpress Microarray databases. The survival data of LIHC cohort was achieved from TCGA Pan-Cancer Clinical Data Resource (TCGA-CDR),^20^ and data of CHCC and LICA were obtained from the supplementary files of corresponding publications.^13, 14^ Survival information of microarray-based cohorts were obtained from corresponding publications.

**Immunotherapy cohorts**

Expression data as well as corresponding clinical data of three immunotherapy-treated cohorts, including Van Allen (melanoma),^21^ Nathanson (melanoma)^22^ and Homet (urothelial cancer) (GSE111636) cohorts, were included for this analysis. Only pretreatment samples were included in this study. Patients in Van Allen and Nathanson cohorts were treated with anti-CTLA-4 treatments, while patients in Homet cohort were treated with anti-PD-1 treatments. Patients who achieved a complete response (CR) or partial response (PR) were categorized as responders, while those who displayed stable (SD) or progressive disease (PD) were defined as non-responders.

**Functional similarity analysis**

Functional similarity (FS) scores between gene pairs were calculated based on the semantic similarities in molecular function (MF) terms of the gene ontology (GO) database. Semantic similarities in MF were measured based on the GO topological structure through using *GOSemSim* package.^23^ Higher resultant score between gene pairs indicates higher functional interactions.

**Statistical analysis**

Statistical analyses and graphical visualization were performed in R software version 4.0.5 (<https://cran.r-project.org/>). Correlation between two continuous variables was determined by Pearson’s r correlation analysis.


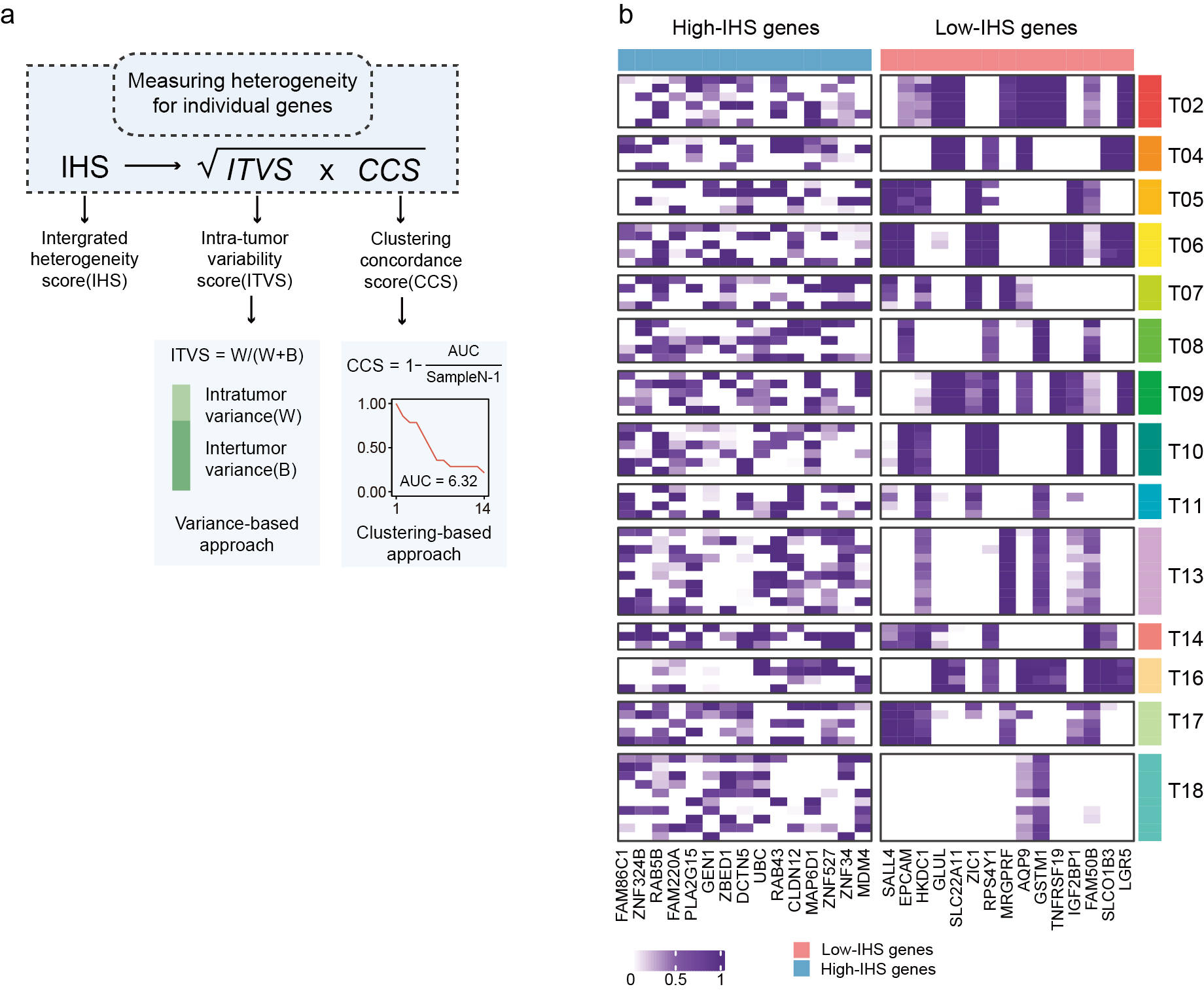


Figure. S1.

(**a**) Schematic presentation of the calculation of integrated heterogeneity score (IHS). (**b**) Heatmap showing the top 15 genes with lowest and highest IHS.


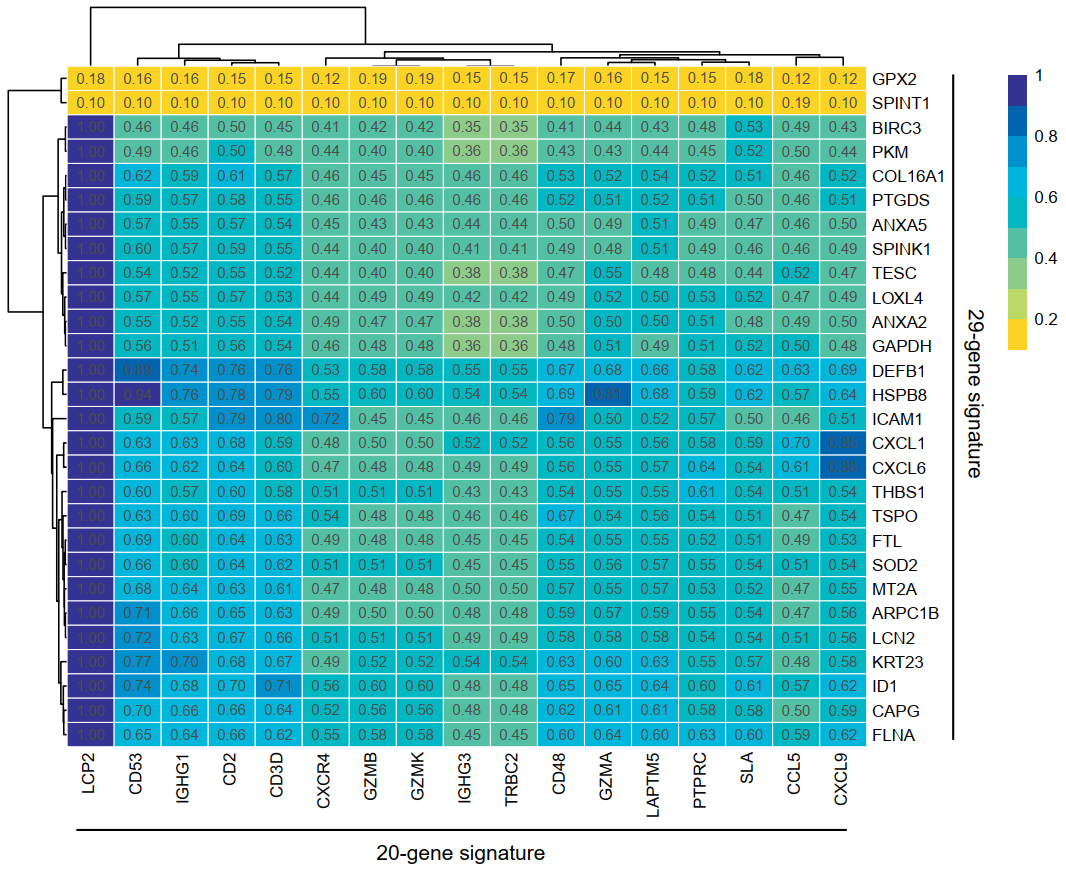


Figure. S2.

Functional similarities of genes between 20-gene and 29-gene signatures. Higher score between gene pairs indicates higher functional interactions.


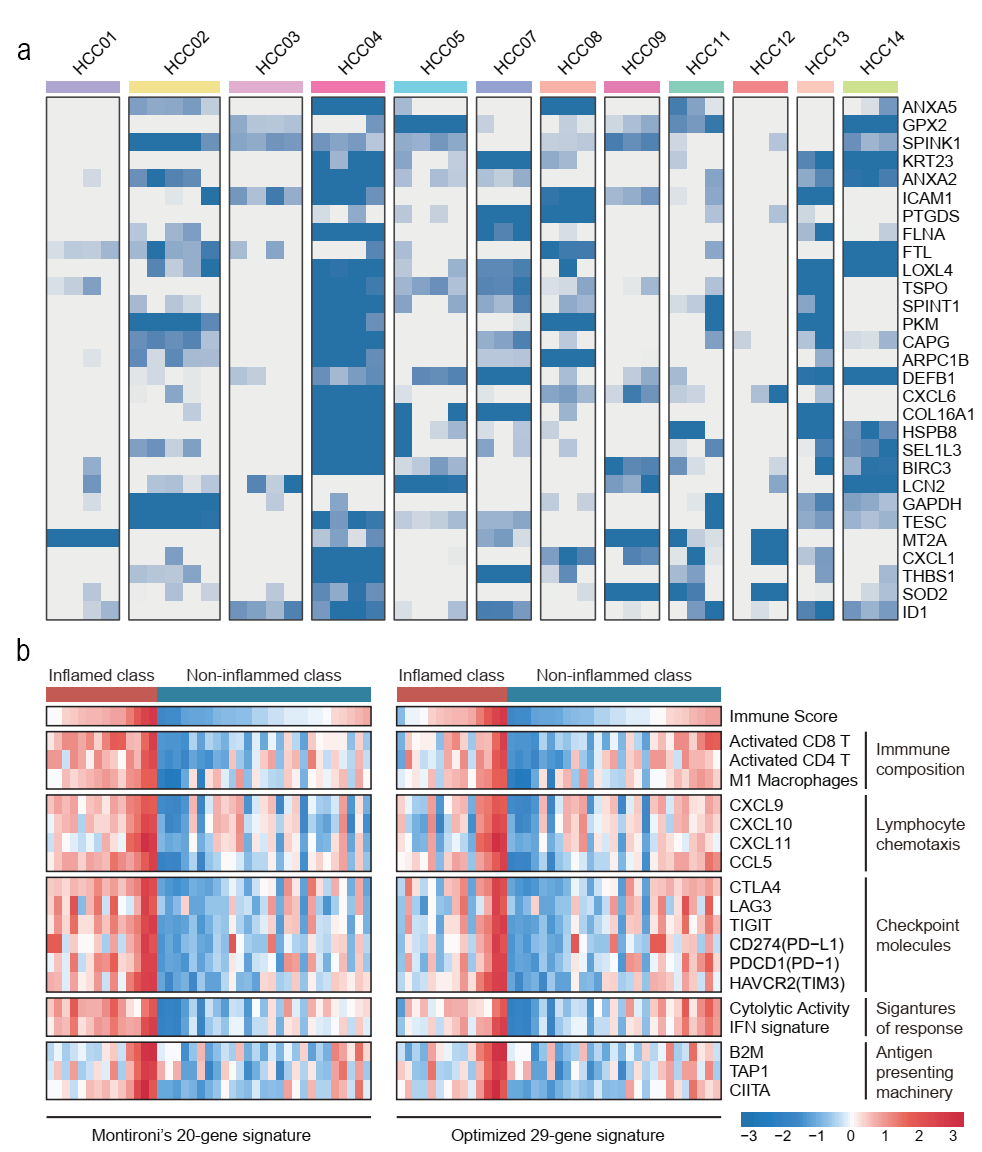


Figure. S3.

(**a**) Expression pattern of genes in the 29-gene signature in GSE136711 cohort. (**b**) Heatmap describing the molecular and immune characteristics of inflamed and non-inflamed HCC classes determined by 29-gene signature or 20-gene signature in GSE136711 cohort.


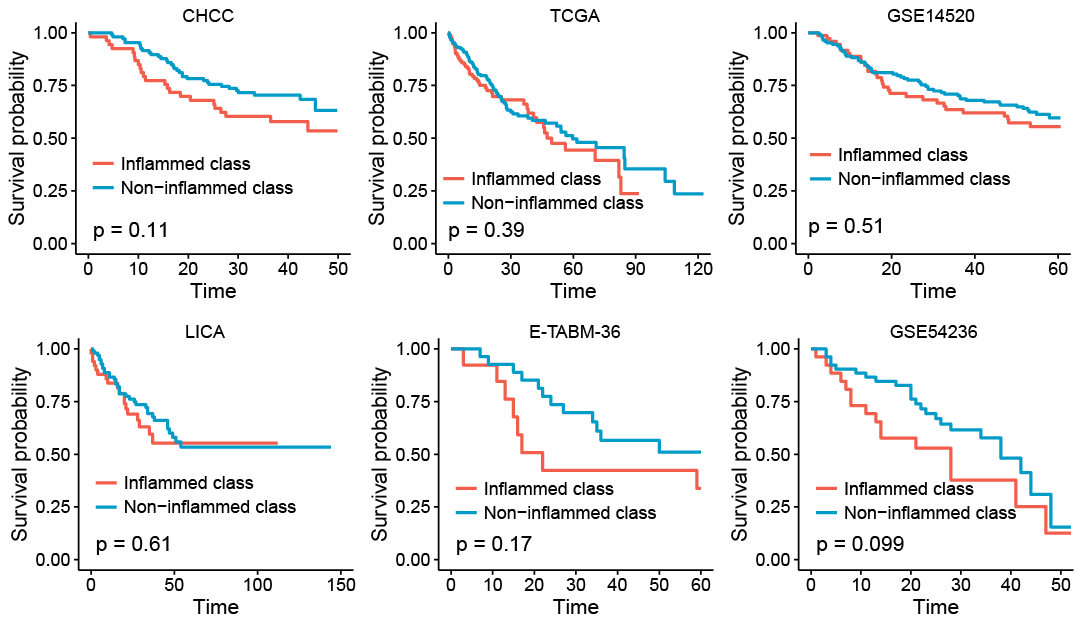


Figure. S4.

Prognostic performance of 29-gene signature in six HCC cohorts, including three sequencing cohorts (TCGA-LIHC, CHCC-HBV and LICA-FR) and three microarray cohorts (GSE14520, GSE54236 and E-TABM-36).


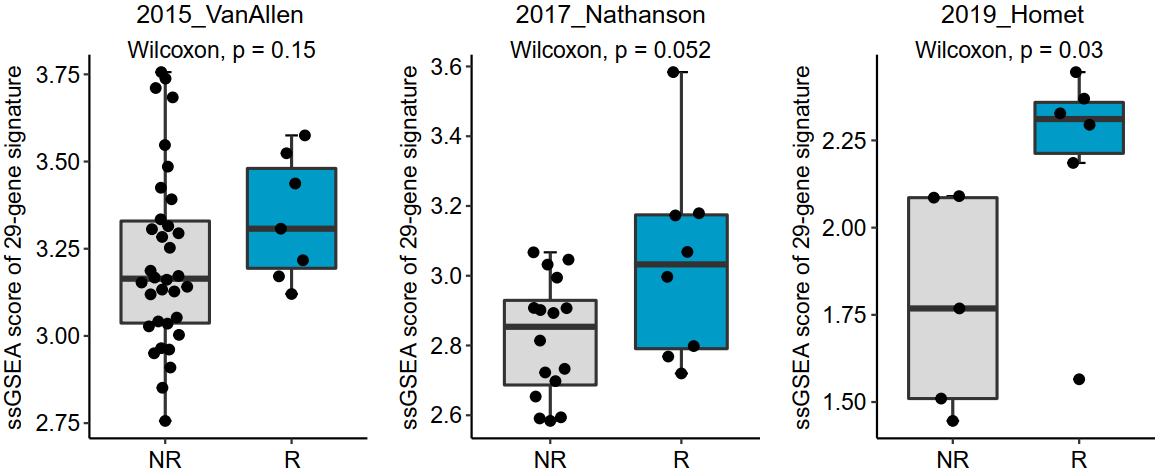


Figure. S5.

Performance of immunotherapeutic prediction of 29-gene signature in three cohorts, including Van Allen (melanoma), Nathanson (melanoma) and Homet (urothelial cancer) cohorts.

**References**

1. Kim D, et al. Graph-based genome alignment and genotyping with HISAT2 and HISAT-genotype. *Nat Biotechnol* **37**, 907-915 (2019).

2. Liao Y, Smyth GK&Shi W. The Subread aligner: fast, accurate and scalable read mapping by seed-and-vote. *Nucleic Acids Res* **41**, e108 (2013).

3. Shen YC, et al. Reliability of a single-region sample to evaluate tumor immune microenvironment in hepatocellular carcinoma. *J Hepatol* **72**, 489-497 (2020).

4. Bachtiary B, et al. Gene expression profiling in cervical cancer: an exploration of intratumor heterogeneity. *Clin Cancer Res* **12**, 5632-40 (2006).

5. Yan W, et al. Three-dimensional mRNA measurements reveal minimal regional heterogeneity in esophageal squamous cell carcinoma. *Am J Pathol* **182**, 529-39 (2013).

6. Dunne PD, et al. Cancer-cell intrinsic gene expression signatures overcome intratumoural heterogeneity bias in colorectal cancer patient classification. *Nat Commun* **8**, 15657 (2017).

7. Biswas D, et al. A clonal expression biomarker associates with lung cancer mortality. *Nat Med* **25**, 1540-1548 (2019).

8. Lee DD&Seung HS. Learning the parts of objects by non-negative matrix factorization. *Nature* **401**, 788-91 (1999).

9. Yoshihara K, et al. Inferring tumour purity and stromal and immune cell admixture from expression data. *Nat Commun* **4**, 2612 (2013).

10. Hänzelmann S, Castelo R&Guinney J. GSVA: gene set variation analysis for microarray and RNA-seq data. *BMC Bioinformatics* **14**, 7 (2013).

11. Montironi C, et al. Inflamed and non-inflamed classes of HCC: a revised immunogenomic classification. *Gut* (2022).

12. Sia D, et al. Identification of an Immune-specific Class of Hepatocellular Carcinoma, Based on Molecular Features. *Gastroenterology* **153**, 812-826 (2017).

13. Gao Q, et al. Integrated Proteogenomic Characterization of HBV-Related Hepatocellular Carcinoma. *Cell* **179**, 1240 (2019).

14. Schulze K, et al. Exome sequencing of hepatocellular carcinomas identifies new mutational signatures and potential therapeutic targets. *Nat Genet* **47**, 505-511 (2015).

15. Ally A, et al. Comprehensive and integrative genomic characterization of hepatocellular carcinoma. *Cell* **169**, 1327-1341. e23 (2017).

16. Roessler S, et al. Integrative genomic identification of genes on 8p associated with hepatocellular carcinoma progression and patient survival. *Gastroenterology* **142**, 957-966.e12 (2012).

17. Kim SM, et al. Sixty-five gene-based risk score classifier predicts overall survival in hepatocellular carcinoma. *Hepatology* **55**, 1443-52 (2012).

18. Villa E, et al. Neoangiogenesis-related genes are hallmarks of fast-growing hepatocellular carcinomas and worst survival. Results from a prospective study. *Gut* **65**, 861-9 (2016).

19. Li B&Dewey CN. RSEM: accurate transcript quantification from RNA-Seq data with or without a reference genome. *BMC Bioinformatics* **12**, 323 (2011).

20. Liu J, et al. An Integrated TCGA Pan-Cancer Clinical Data Resource to Drive High-Quality Survival Outcome Analytics. *Cell* **173**, 400-416.e11 (2018).

21. Van Allen EM, et al. Genomic correlates of response to CTLA-4 blockade in metastatic melanoma. *Science* **350**, 207-211 (2015).

22. Nathanson T, et al. Somatic Mutations and Neoepitope Homology in Melanomas Treated with CTLA-4 Blockade. *Cancer Immunol Res* **5**, 84-91 (2017).

23. Yu G, et al. GOSemSim: an R package for measuring semantic similarity among GO terms and gene products. *Bioinformatics* **26**, 976-8 (2010).
